# Case Report: Early Breast Cancer Recurrence Mimicking BIA-ALCL in a Patient With Multiple Breast Procedures

**DOI:** 10.3389/fsurg.2021.606864

**Published:** 2021-03-09

**Authors:** Marco Materazzo, Gianluca Vanni, Marco Pellicciaro, Lucia Anemona, Francesca Servadei, Erika Giacobbi, Andrea Farinaccio, Chiara Adriana Pistolese, Tommaso Perretta, Marcello Chiocchi, Valentina Bruno, Federico Tacconi, Amir Sadri, Adriano De Majo, Camilla Di Pasquali, Rosaria Meucci, Francesca Santori, Maria Cotesta, Oreste Claudio Buonomo

**Affiliations:** ^1^Breast Unit, Department of Surgical Science, Policlinico Tor Vergata University, Rome, Italy; ^2^Anatomic Pathology, Department of Experimental Medicine, Policlinico Tor Vergata University, Rome, Italy; ^3^Department of Cardiothoracic Anesthesia, Tor Vergata University Hospital, Rome, Italy; ^4^Department of Diagnostic Imaging and Interventional Radiology, Molecular Imaging and Radiotherapy, Policlinico Tor Vergata University, Rome, Italy; ^5^Section of Gynecology and Obstetrics, Academic Department of Biomedicine and Prevention, University Tor Vergata, Rome, Italy; ^6^Division of Thoracic Surgery, Department of Surgical Science, Policlinico Tor Vergata University, Rome, Italy; ^7^Plastic Surgery, Great Hormond Hospital for Children NHS Foundation Trust, London, United Kingdom

**Keywords:** breast implant associated-anaplastic large cell lymphoma, breast cancer, locoregional recurrence, macro textured breast implants, residual breast tissue, case report, breast seroma, immediate breast reconstruction

## Abstract

Breast reconstruction plays a fundamental role in the therapeutic process of breast cancer treatment and breast implants represents the leading breast reconstruction strategy. Breast Implant Associated-Anaplastic Large Cell Lymphoma (BIA-ALCL), locoregional recurrence in the skin flap, and skin flap necrosis are well-known complications following mastectomy and immediate breast reconstruction (IBR). We report a case of locoregional cancer recurrence in the mastectomy flap mimicking BIA-ALCL, in a patient who underwent 6 breast procedures in four facilities across 15 years including immediate breast reconstruction with macrotextured breast implants. Despite the rate and onset of the disease, clinicians should be aware of BIA-ALCL. Due to the risk of false negative results of fine needle aspiration, clinical suspicion of BIA-ALCL should drive clinicians' choices, aside from cytological results. In the present case, surgical capsulectomy of the abnormal periprosthesic tissue revealed locoregional recurrence.

## Introduction

Breast reconstruction attained a fundamental role in the therapeutic process of women facing breast cancer on the path to restore the female body image and quality of life (1–3). Regardless of stage and age (4, 5), Breast implants represent the primary breast reconstruction strategy (81.2%) (6).

Despite its popularity, textured breast implants were associated with the onset of Breast Implant Associated-Anaplastic Large Cell Lymphoma (BIA-ALCL), a rare form of T-cell Lymphoma (7). In this perspective, macrotextured breast implants were retired from the market in 2019 due to the risk of the BIA-ALCL (8, 9). BIA-ALCL clinical presentation is represented by unilateral late peri-implant cold seroma containing malignant cells, or less commonly as a mass attached to the breast implants with or without regional lymph node involvement (7). With a median onset of 8 years from implants introduction, BIA-ALCL incidence is difficult to calculate due to the unknown number of women with breast implants worldwide (10).

Another complication of mastectomy and immediate breast reconstruction (IBR) is represented by locoregional recurrence in the skin flap (11). Diversely, skin flap necrosis is a well-known complication after IBR, occurring in 5–30% of cases (12). Meticulous surgical technique is required in order to reduce the risk of this complication (12). Achieving the optimal balance between oncologic outcome and skin necrosis requires a resection radical as possible without compromising the skin flap viability (13).

Herein, we report a case of locoregional breast cancer recurrence in the mastectomy flap mimicking BIA-ALCL, in a patient who underwent prophylactic mastectomy with immediate reconstruction with macrotextured breast implants. The Institutional Review Board PTV: Policlinico Tor Vergata University waived the need for a formal approval for the clinical reports. The work was conducted in accordance with the Declaration of Helsinki. Written informed consent was obtained from the patient for publication of this case report and the accompanying images. This work is reported by following the CARE guidelines.

## Case Report

A 51-years-old G2P1 smoker (31 packs-year) post-menopausal woman was admitted as an outpatient to our facilities with a breast cancer diagnosis in the skin flap of previous mastectomy. Family history was positive for breast cancer (mother) and brain cancer (brother). Past medical history enlisted previous diagnoses of depression under medical treatment and hysterectomy in 1989 due to miscarriage.

Breast history reported a previous lumpectomy for a benign phyllodes tumor in 2006 in another facility. Subsequently, in 2011, in a second different facility and during postoperative follow-up, the patient underwent fine-needle aspiration which was classified as suspicious for malignancy (C4). Wire-guided lumpectomy demonstrated Flat Epithelial Atypia, adenosis, apocrine metaplasia, and ductal papillomatosis.

At the end of the same year, due to the increase of broad microcalcification in the left breast and positive family history, the patient underwent a bilateral prophylactic Nipple Sparing Mastectomy (NSM) with implants of macrotexured Allergan 410 MF 420 g breast implants. Both breast specimens revealed the absence of cancer. Postoperative course reported severe nipple-areola necrosis with full-thickness skin necrosis that required two-time surgical revision and left breast implant substitution. Then, due to the poor aesthetic result, the patient decided to undergo a bilateral prosthesis removal in a third facility and refused further breast reconstruction.

Following the breast implants removal, the patient continued postoperative follow-up. Eventually, in January 2020, the patient went through outpatient evaluation after detecting evidence of left breast mass in the retroareolar residual tissue. Ultrasound guided biopsy revealed the presence of an Invasive ductal carcinoma. After surgical and psychiatric outpatient evaluation, the patient agreed to undergo a bilateral reconstruction. The patient underwernt a left breast lumpectomy plus sentinel lymph node biopsy, a right retroareolar breast tissue remnant removal and a bilateral positioning of breast tissue expander with methylene blue. The left breast mass revealed an invasive ductal carcinoma G1 with a low grade ductal carcinoma *in situ*. Estrogen receptor (ER) positive 95%, Progesterone receptor (PR) positive 95%, Ki67 10%, and c-Erb-B2 negative score (HER2 score) 0. Sentinel lymph node biopsy revealed four lymph nodes, two of which were proven micrometastatic and 1 with isolated tumor cells. The right breast remnants didn't reveal any breast lesion. The postoperative course was regular and no breast modified Clavien-Dindo ≥2 complications were reported (14).

However, during postoperative follow up 6 months after the operation, the patient reported left breast discomfort, swelling, and green urinary output as seen in a breast implant rupture. Preoperative fine needle aspiration of the seroma didn't demonstrate any atypical cell. Prior surgery, magnetic resonance imaging was not performed due to the previous breast tissue expander insertion. Patient was admitted to the hospital for a left breast tissue expander substitution on July 3rd,2020.

**Figure 1:**
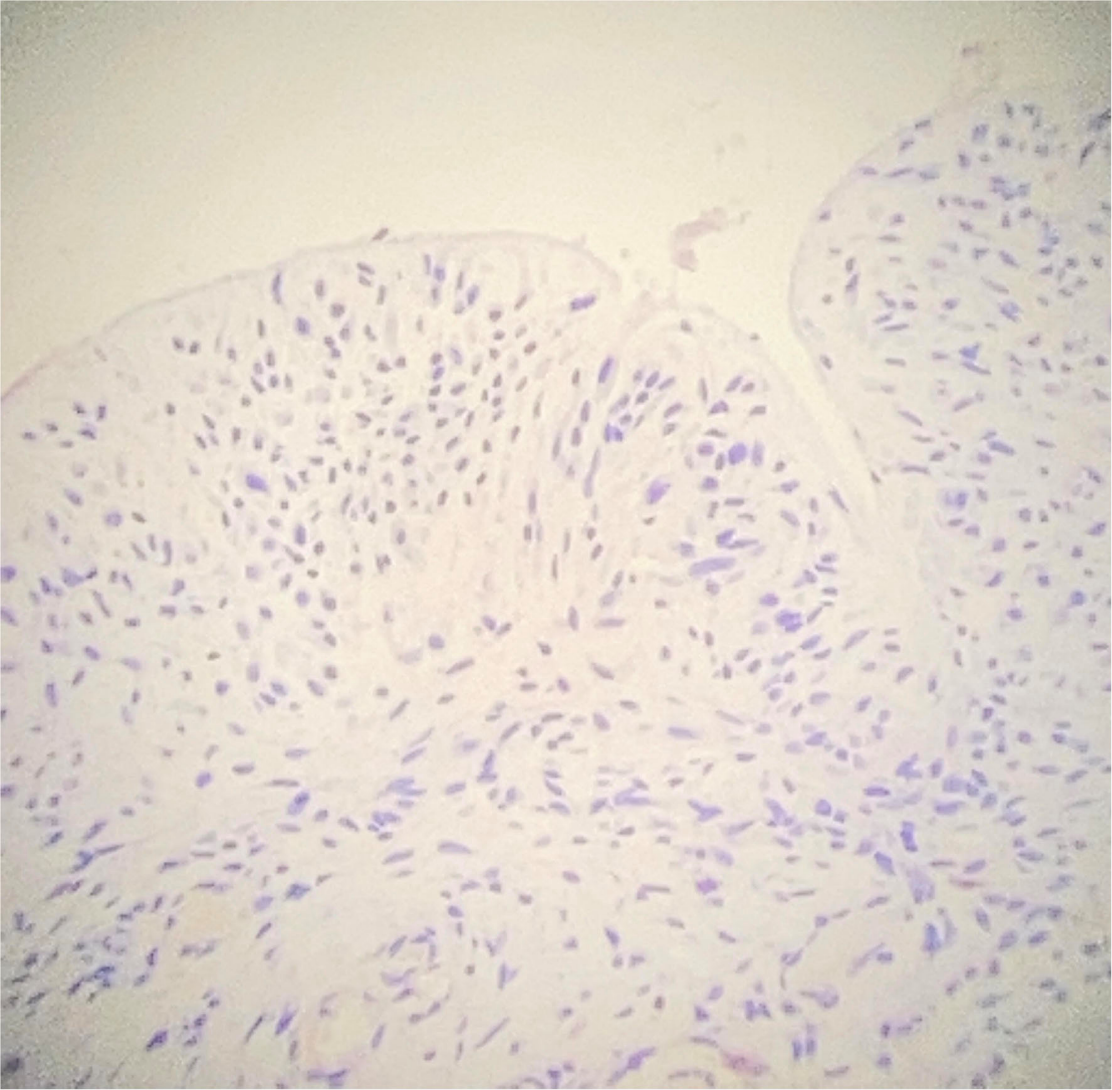
Immunohistochemical (IHC) study of periprothestic staining. CD30 negative staining on periprosthetic capsule (original magnification 10×).

**Figure 2:**
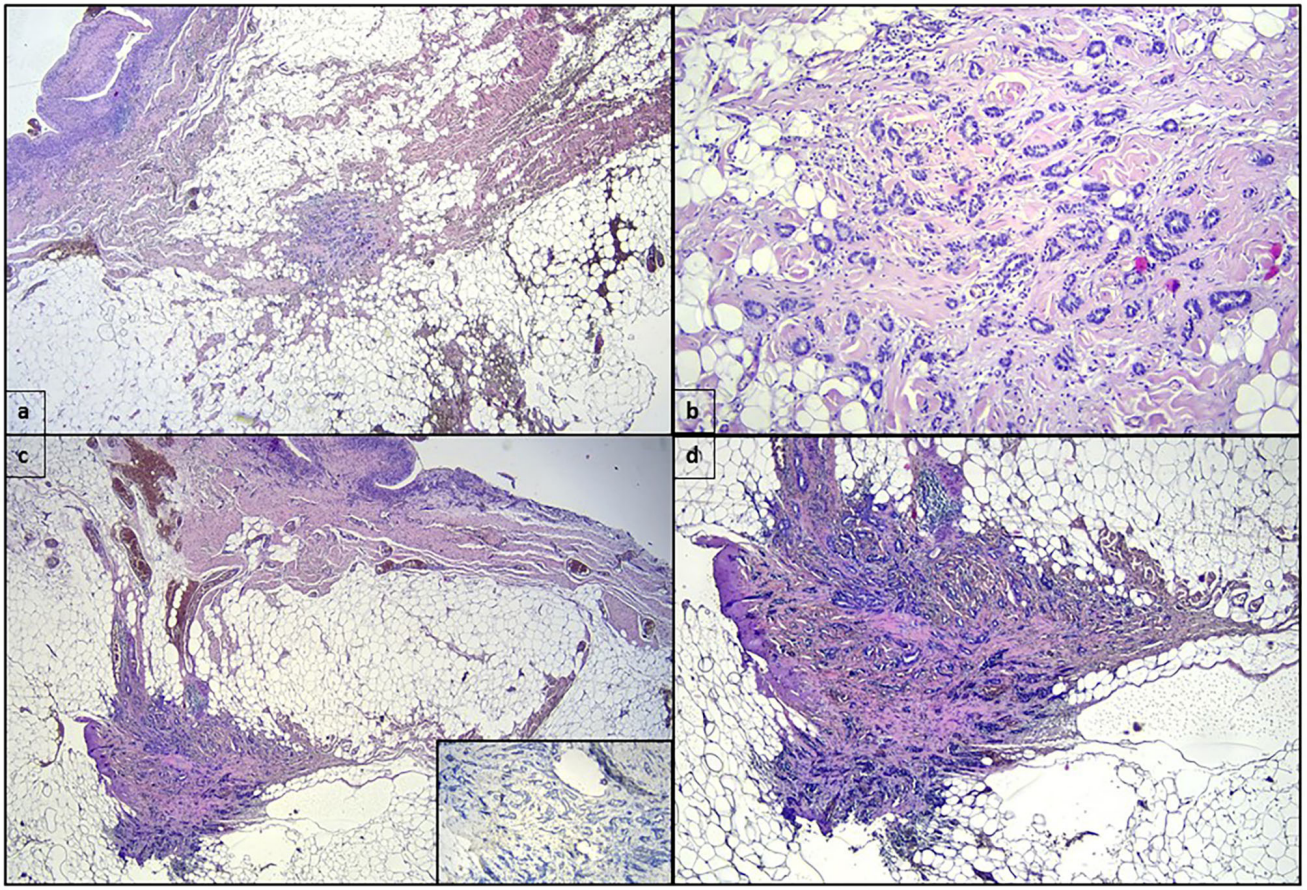
**(a)** One mm-wide invasive ductal carcinoma in the fibroadipose tissue surrounding the periprosthetic capsule, whose inner surface can be recognized in the upper left of the panel (hematoxylin eosin, original magnification 2×). **(b)** Higher magnification highlights the invasive ductal carcinoma shown in **(a)** (hematoxylin eosin, original magnification 10×). **(c)** Another section of the fibroadipose tissue surrounding the periprosthetic capsule showing a second focus of invasive ductal carcinoma 2 millimeters-wide. The insert shows the absence of myoepithelial layer in the ductal structures (hematoxylin eosin, original magnification 2×; immunostaining for p63, clone Leica, 10× in the insert). **(d)** Higher magnification of invasive ductal carcinoma showed in **(c)** (hematoxylin eosin, original magnification 4×).

Surgical exploration revealed periprothesic breast tissue thickening and yellow citrine seroma without rupture of breast implant tissue expander, mimicking BIA-ALCL symptoms. During the surgical procedure, periprothesic tissue specimen and periprosthetic fluid effusion were collected and sent to cytological and microbiological evaluation. The periprostetic fluid and periprothesic didn't reveal bacterial colonization, nor the presence of atypical cell (Figure 1). The periprothesic breast tissue revealed several homolateral infiltrative breast cancer recurrence nidus in the fibroadipose tissue, as shown in Figure 2.

## Discussion

The postoperative onset of BIA-ALCL is an emerging problem in breast reconstructive surgery (7, 15). Breast cancer patients may experience increased levels of anxiety and or depression (16, 17). Media coverage could have given rise to anxiety in breast cancer patients who are planned to undergo implant-based reconstruction (15). Common clinical symptoms include abrupt late cold seroma or periprothestic mass after several years (7–10) from breast implant insertion (7, 9) with a higher reported rate of occurrence in patients with macrotexured implants (8). With a median onset of 8 years from implants introduction, BIA-ALCL cases has been reported even after macrotextured breast implant substitution with smooth prosthesis (7). Despite the low incidence of the disease, in our clinical case, positive clinical history for macrotexured Allergan 410 MF 420 g breast implant 9 years previously and early intraoperative abnormal thickness of periprothesic tissue were consistent with BIA-ALCL diagnosis.

BIA-ALCL current management consists of breast ultrasound and magnetic resonance imaging (10). Ultrasound represents the first choice for evaluating effusion volumes, breast mass dimensions and for obtaining a large amount of fluid (at least 10 mL, but ideally 50 ml) (18, 19). Magnetic resonance imaging provides useful information as the presence of a mass or axillary lymphadenopathy (18). In our clinical case, magnetic resonance imaging was not performed due to device manufacturers recommendation (20). Despite the increased awareness due to media coverage in recent years (15), current imaging appears suboptimal in BIA-ALCL detection (18) and requires assessment in a tertiary breast cancer facility (21, 22). Correct evaluation of late seroma is challenging (19, 23). In literature at least a case of BIA-ALCL with negative aspiration cytology is reported in literature (23). Conversely, up to 0.8% patients with macrotextured breast implants could develop seroma during the follow up period (24), and it was calculated that <5% of delayed seromas may represent BIA-ALCL (23).

However, an additional complication of mastectomy plus IBR, as seen in our patient, is represented by locoregional recurrence of breast cancer (11). Clinicians should aspire to maintain the rate of locoregional recurrence below 5%, utilizing post-mastectomy radiotherapy (PMRT) (25).

Lymphatic spread, metastasis caused by tumor seeding, and incomplete tumor removal are described in the literature as a cause of locoregional recurrence following mastectomy (26). However, another cause of locoregional recurrence may be linked to residual breast tissue in the superficial margins, which could potentially promote metachronous breast cancer (27). Breast carcinoma in residual breast tissue following prophylactic bilateral procedure is described in the literature and the personal risk appears to be linked with familial and personal history, as recorded in our patient (26, 28). Moreover, several cases of Nipple discharge in women with previous NSM with or without pregnancy are reported in the literature supporting this theory (29, 30).

In our clinical case, several different breast surgical procedures in different facilities could have partially affected the risk of locoregional recurrence. Kesson et al. reported a reduction of 18% in breast cancer mortality at 5 years and 11% in all-cause mortality at 5 years when patients were treated by a multidisciplinary team (31). The modern approach of breast treatment and follow up is headed by the knowledge of breast cancer etiology (32, 33) and the subsequent development of systemic tailored strategy in a multidisciplinary setting (4, 31).

In this particular clinical case, the patient underwent six breast procedures in four facilities across 15 years and in our opinion, depression may have played a pivotal role in the decision making process. Depression is a well-known risk factor for poor medication-taking behavior, adherence to treatment and reduced survivorship among breast cancer patients (34). To avoid any detrimental effect on the clinical outcome, we routinely offer a preoperative psychological evaluation for any breast cancer patient. In addition to reduced patient mortality, treatment in a centralized tertiary facility reduces the number of unnecessary interventions (35), reduces the hospitalization of patients (36–38), and provides a lower rate of complications during reconstruction surgery (1) or innovative approach to reduce the impact of surgery (36, 39). In our opinion, tertiary breast cancer facility could produce a higher medical standard for patients and could provide a better medical treatment for breast pathology.

## Conclusion

The postoperative onset of BIA-ALCL is an emerging problem in breast reconstructive surgery (7, 15). Despite the low incidence and late onset of the disease, clinicians should be aware of this rare entity. Due to the risk of false negative results of fine needle aspiration, surgeons should carefully perform surgical exploration to avoid under treatment and misdiagnosis. Clinical suspicion of BIA-ALCL should drive clinicians' choices, aside from cytological results. In the present case, surgical capsulectomy of the abnormal periprosthesic tissue revealed locoregional recurrence. Psychiatric disorder represents a risk factor for adherence to treatment and reduced survivorship. Psychological assessment should be routinely provided in tertiary breast cancer facilities.

## Data Availability Statement

The raw data supporting the conclusions of this article will be made available by the authors, without undue reservation.

## Ethics Statement

Written informed consent was obtained from the individual(s) for the publication of any potentially identifiable images or data included in this article.

## Author Contributions

MM and MP prepared the manuscript. MC, FS, AD, and CD acquired the data. EG, FS, and LA performed histological examination. VB, FT, and AS performed data analysis. AF, CP, TP, and MP performed interpretation of external imaging. RM, GV, and OB performed review of the literature. All authors contributed to the article and approved the submitted version.

## Conflict of Interest

The authors declare that the research was conducted in the absence of any commercial or financial relationships that could be construed as a potential conflict of interest.
